# Engineering T Cell Development for the Next Generation of Stem Cell-Derived Immunotherapies

**DOI:** 10.1089/genbio.2023.0008

**Published:** 2023-04-18

**Authors:** Yale S. Michaels, Lauren J. Durland, Peter W. Zandstra

**Affiliations:** ^1^ School of Biomedical Engineering, University of British Columbia, Vancouver, Canada; University of British Columbia, Vancouver, Canada.; ^2^ Department of Biochemistry and Medical Genetics, Rady Faculty of Health Sciences, University of Manitoba, Winnipeg, Canada; University of British Columbia, Vancouver, Canada.; ^3^ CancerCare Manitoba Research Institute, CancerCare Manitoba, Winnipeg, Canada; and University of British Columbia, Vancouver, Canada.; ^4^ Michael Smith Laboratories, University of British Columbia, Vancouver, Canada.

## Abstract

Engineered T cells are at the leading edge of clinical cell therapy. T cell therapies have had a remarkable impact on patient care for a subset of hematological malignancies. This foundation has motivated the development of off-the-shelf engineered cell therapies for a broad range of devastating indications. Achieving this vision will require cost-effective manufacturing of precision cell products capable of addressing multiple process and clinical-design challenges. Pluripotent stem cell (PSC)-derived engineered T cells are emerging as a solution of choice. To unleash the full potential of PSC-derived T cell therapies, the field will require technologies capable of robustly orchestrating the complex series of time- and dose-dependent signaling events needed to recreate functional T cell development in the laboratory. In this article, we review the current state of allogenic T cell therapies, focusing on strategies to generate engineered lymphoid cells from PSCs. We highlight exciting recent progress in this field and outline timely opportunities for advancement with an emphasis on niche engineering and synthetic biology.

## Introduction

For the past decade, there has been tremendous progress in treating cancer with cellular immunotherapies. In particular, chimeric antigen receptors (CARs) that redirect T cells to recognize CD19 or BCMA have shown remarkable efficacy against certain types of leukemia, lymphoma, and multiple myeloma.^1^ So far, six CAR-T therapies have been approved by the U.S. Food and Drug Administration (FDA) since 2017, with several hundred more products at various stages of clinical development.^2^ CAR-T cells are also being explored for a multitude of nononcology indications, including transplant rejection, infection, autoimmunity, cardiovascular disease, fibrosis, and senescence.^3–7^

Despite their impressive and expanding resume, several technical hurdles must be overcome before T cell therapies realize their full potential. Questions of safety, efficacy, antigen escape, and translation to solid tumors are important challenges that have been extensively reviewed elsewhere.^8^ Another major bottleneck is the manufacturing process itself. The predominant method of manufacturing T cell therapies today is to source T cells from the patient themselves, genetically engineer them to recognize malignant cells using bespoke viral transduction pipelines and infuse the modified cells back into the patient.^1,9^ This personalized manufacturing process adds significant cost^10,11^ and variability^12,13^ to T cell therapies. Building more robust, scalable, and reproducible manufacturing workflows for T cell therapies will help improve product safety and efficacy and will expand access to these lifesaving therapies.^9^

In this review, we examine the current state of allogenic T cell therapies, paying particular attention on strategies to generate engineered lymphoid cells from pluripotent stem cells (PSCs). We also discuss recent progress in the field and consider opportunities for advancement, especially with regard to niche engineering and synthetic biology.

### Challenges of autologous CAR-T therapies

Unlike small molecules and biologics, which are produced in scalable, controllable, and fully defined environments inside chemical or biological reactors (Domokos, Nagy, 2021), T cell therapy products are living medicines. Currently, for clinical use, T cell therapies are predominantly produced in single doses, sourcing the unmodified T cells from the patient themselves.^9,14^

These autologous T cell therapies face several significant manufacturing challenges. One is cost. For allogenic products, each time a patient is prescribed CAR-T, a personalized multistep workflow is initiated.^9^ The patient donates peripheral blood and their T cells are isolated. Next, the T cells are stimulated *ex vivo* and begin to undergo massive expansion. Once the T cells have been activated, they are transduced with a viral vector comprising the CAR transgene. Finally, the CAR-T product undergoes release testing for quality assurance before being infused into the patient.

This entire workflow, including production of the CAR viral vectors, is carried out in compliance with good manufacturing practices. Because of this complex personalized production process, the price of CAR-T is remarkably high compared with other types of therapies, with one dose priced on the order of $500,000.^10,11,15^ Whether the benefits of CAR-T therapies to patients can justify their current price tag is difficult to quantify and a matter of ongoing debate.^11,16^ Not only do excessive costs limit the number of patients who are able to access currently approved therapies, but they also restrict the scope of future indications for which CAR-T cells are contemplated.

A second challenge is variability in product quality. This variability exists in multiple aspects. There is donor-to-donor variability in the number and quality of T cells available for producing the CAR-T product.^12,13,17^ Beyond the biological diversity that exists among individuals in the healthy population, this problem is exacerbated by the impact of cancer on a patient's T cell compartment. Radiation and chemotherapy, the first-line treatments for most cancers, diminish the number and functionality of a patient's T cells.^18–20^ Consequently, a meaningful percentage of patients are not able to produce a graft of good enough quality to proceed to infusion.^21^

In addition to donor-to-donor variability, there is also heterogeneity within an individual graft. Depending on the specific manufacturing process, the isolated and expanded T cells comprise a mixture of T cell subtypes at various states of polarization and differentiation along the effector-memory axis.^22^ Furthermore, not all T cells in the graft will successfully be transduced with the CAR, leading to a mixture of modified and unmodified cells. Even among modified cells, the semirandom nature of retroviral vector integration leads to heterogeneity in expression level that impacts functionality and carries the risk that important loci are disrupted.^23^ As grafts comprise billions of cells, even rare oncogenic events must be considered.

### Alternatives to autologous T cell therapies

Cost, variability, and product quality control are widely recognized barriers to expanding on the success of CAR-T therapy. Hereunder, we discuss how gene-edited CARs and allogeneic T cell products can partially address these challenges. We also explore the advantages and limitations of *in vivo* CAR transduction as a low-cost alternative to *ex vivo* manufacturing. We then make the case for PSC-derived T cell products as a compelling long-term solution for accessible high-performance immunotherapies.

One way to avoid the gene expression heterogeneity caused by semirandom viral integration is to target the CAR to a defined locus using precision gene editing such as CRISPR-Cas9. In a preclinical model of B cell leukemia, introducing a CD19 CAR into the T cell receptor alpha chain constant (TRAC) locus with CRISPR-Cas9 reduced both median CAR expression and expression variability compared with a virally engineered control.^23^ TRAC knock-in also significantly improved tumor clearance compared with virally modified controls. Virally introduced CARs are typically expressed at much higher levels than endogenous T cell receptors. High constitutive CAR expression can drive T cells into an exhausted state and impair their functionality.^23^ In contrast, knocking a CAR into the TRAC locus leads to a more T cell receptor (TCR)-like expression level, mitigating exhaustion and improving efficacy.^23^

Gene-edited CARs may provide a more consistent and effective product. However, further improvements are required. Although recent designer fusion proteins can improve CRISPR-Cas9 knock-in efficiencies, integration rates for large multikilobase payloads remain low in primary T cells.^24^ CRISPR is also prone to off-targets mutagenesis, meaning edited products will still comprise a mix of correctly modified, incorrectly modified, and unmodified cells.^25^ Precise editing workflows, especially ones that require both nucleofection and AAV homology donors will add cost to the manufacturing process, although this can be mitigated using nonviral editing.^26,27^ Gene-edited CAR-T cells will not solve the problem of intradonor heterogeneity.

Primary donor-derived allogeneic T cell therapies can partially address the challenge of donor–donor variability. By sourcing T cells from healthy donors, it is possible to circumvent the negative impact that cancer and cancer therapies have on a patient's T cell compartment. Although autologous CAR-T products often fail release testing,^28^ in recent trials of primary allogeneic CAR-T therapy, all enrolled patients were able to receive treatment—none were excluded because of poor-quality T cell products.^29,30^ Allogeneic therapies may also simplify the manufacturing process as cell therapy products can be cryopreserved and utilized on demand, reducing the time delay required for autologous production.^30^

Despite their advantages, primary allogeneic therapies must overcome immunological barriers between the donor and recipient. Graft versus host reactivity is an important safety concern and avoidance of graft rejection to achieve a durable therapeutic benefit is an added challenge.^29^ Although healthy donors can provide a larger number of functional T cells compared with cancer patients, there is still sizable heterogeneity in T cell abundance and functionality across healthy individuals.^31^ Beyond the brief overview provided earlier, the advantages and limitations of primary allogeneic therapy are more thoroughly reviewed elsewhere.^32^

Gene-edited T cells and allogeneic donors can theoretically reduce variability in CAR-T outcomes, but neither approach will eliminate the costly *ex vivo* T cell manufacturing process. A potential cost-saving solution is to perform a rapid *ex vivo* transduction step and allow the lengthy expansion process to occur within the patient. In one ongoing clinical trial of this approach, an extremely rapid *ex vivo* manufacturing step (∼2 days) has shown promising early results.^33^

Beyond shortening and automating the CAR-T manufacturing processes, an elegant alternative is to eliminate the *ex vivo* cell therapy manufacturing step altogether by delivering the CAR to T cells within a patient's body. This has previously been achieved by infusing mice with CAR expression cassettes using delivery systems such as nanoparticles loaded with DNA or mRNA,^34,35^ or viral vectors.^36,37^ A recently proposed hybrid approach involves taking patient T cells, seeding them in an alginate scaffold along with viral particles and T cell activating reagents, and implanting the biomaterial back into the patient where the cells become transduced and expand *in vivo.*^38^ These *in vivo* and hybrid manufacturing processes should reduce costs, time, and labor associated with CAR-T manufacturing if they prove to be effective in humans. Nevertheless, these approaches carry additional risks, including the possibility of transducing off-target cell types.

Primary allogeneic CAR-T cells can help address product variability, and *in vivo* CAR delivery may reduce manufacturing costs. PSCs are an extremely attractive alternative approach because they can simultaneously address both challenges. Rather than obtaining T cells from individual donors, they can be differentiated from PSCs (PSC allogeneic CAR-T). PSCs are amenable to clonal expansion and unlimited self-renewal.^39^ This means CARs, or other therapeutic augmentations, can be easily be introduced at targeted loci through gene editing.^40^

After rigorous quality control, clones that contain the desired modification and are free from off-target mutations and chromosomal abnormalities can be expanded at scale in bioreactors for subsequent *in vitro* differentiation into T cells. The resulting cell therapy product can be cryopreserved for on-demand off-the-shelf therapy.^41^ PSCs have the potential to serve as a low-cost source of high-quality clonally engineered T cell therapies. To realize this vision and enable large-scale manufacturing of T cells for therapy, researchers have worked to develop and improve PSC-to-T cell differentiation processes.

## Efforts to Make PSC-Derived T Cells

PSC differentiation protocols are often designed using insights from embryogenesis; therefore, a thorough understanding of T cell development as it occurs *in vivo* is critical to the success of efforts to generate PSC-derived T cells. In this section, we briefly outline the process of T cell development, then highlight how our understanding of this process has shaped the development of *in vitro* T cell differentiation protocols. More comprehensive descriptions of T cell development and hematopoiesis can be found in several excellent reviews.^42–47^

### T cell development *in vivo*

All cells of the hematopoietic system are derived from a subset of mesoderm. During early embryogenesis, pluripotent cells ingress through a transient structure called the primitive streak, where they receive signals that prime them toward the hematopoietic lineage, forming the so-called hematopoietic mesoderm compartment.^48^ The hematopoietic mesoderm gives rise to at least two distinct waves of hematopoietic progenitors, which are distinguished *in vivo* based on where and when they emerge, as well as the differentiated cell types they produce. The “primitive” wave is the first to emerge, and is first detected at E7 in mouse^49^ and day 18.5 of gestation in human.^50^ Progenitors of the primitive wave are derived from “blood islands,” or clusters of hematopoietic and endothelial cells in the yolk sac. This early wave of hematopoiesis is transient, and gives rise to a small subset of hematopoietic cell types, including erythrocytes, megakaryocytes, and macrophages.^49^

Importantly, the primitive waves do not produce any cells of the lymphoid lineage, including T cells. In contrast, the “definitive” wave of hematopoiesis emerges later in developmental time, at E11.5 in mouse^51^ and CS14 in human.^52^ Definitive hematopoietic progenitors are derived from endothelial cells with hematopoietic potential, or hemogenic endothelial (HE) cells, within the embryo proper, predominantly in the aorta-gonad-mesonephros region, which undergo endothelial to hematopoietic transition (EHT) to generate hematopoietic cells. In contrast to the apparently restricted potential of primitive progenitors, the definitive wave gives rise to hematopoietic stem cells (HSCs), or single cells that can reconstitute the entire blood system upon transplantation into a new host.^51^

In recent years, the definitions of primitive versus definitive hematopoiesis have become blurred. For instance, studies in mouse have identified yolk sac-derived hematopoietic progenitors with lymphoid potential,^53–57^ and corresponding populations have been described in studies using human PSCs.^58^ In addition, intraembryonic hematopoiesis does not exclusively produce HSCs, as nonengrafting multipotent progenitors, as well as bi- and unipotent progenitors derived from the embryo proper before HSC emergence have been described by multiple independent groups.^59–62^ Although researchers have noted that certain T cell subsets are preferentially derived from progenitors that emerge before HSC development,^63^ functional differences between the T cells derived from yolk sac progenitors, intraembryonic progenitors, and HSCs have not yet been characterized.

**FIG. 1.**
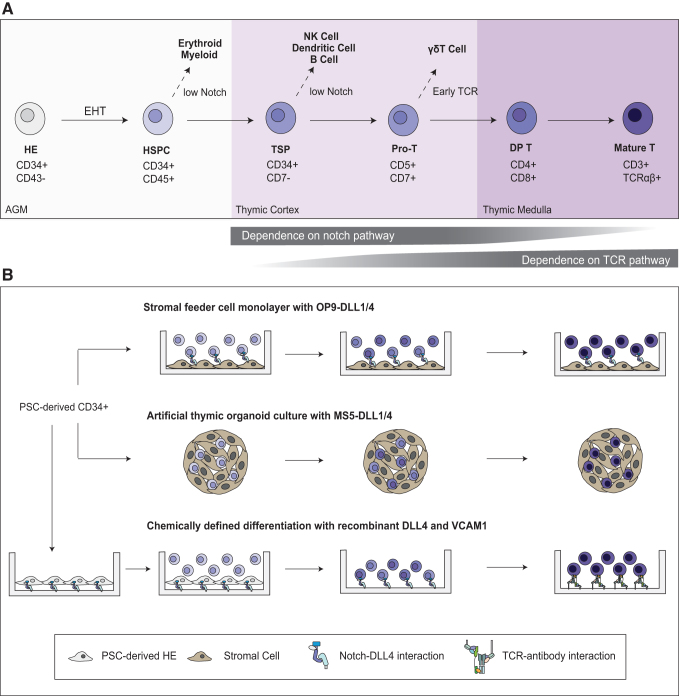
Using developmental biology to guide *in vitro* T cell differentiation. **(A)** Top: Schematic outline of T cell development in the human thymus. Bottom: Relative importance of Notch and TCR signaling during T cell development. **(B)** Feeder-based and chemically defined T cell differentiation protocols. PSC-derived CD34+ HE cells or HSPCs are cultured on immortalized stromal cells expressing Notch ligands to promote T cell differentiation in monolayer cultures. PSC-derived CD34+ HSPC cells are incorporated into spheroids with stromal cells expressing Notch ligands to promote T cell differentiation in artificial organoid cultures. In chemically defined stepwise differentiation protocols, PSC-derived CD34+ cells are cultured on plates coated with DLL4 and VCAM1, with different chemically defined media to induce EHT, Pro-T, and DP T cell development, respectively. DP cells are then exposed to nonspecific TCR stimulation to promote further maturation to the CD8SP stage. CD8SP, CD8 single positive; DP, double positive; EHT, endothelial to hematopoietic transition; HE, hemogenic endothelium; HSPC, hematopoietic stem and progenitor cell; PSC, pluripotent stem cell; TCR, T cell receptor; TSP, thymus-seeding progenitors.

Regardless of developmental origin, T-competent progenitors initially seed the thymus, the primary organ responsible for T cell development^64^ (Fig. 1A). The thymus is organized into various anatomical zones with distinct signaling environments; these different signals promote transition to successive developmental stages as T cell progenitors, or thymocytes, migrate through the thymus.^65^ The earliest thymus-seeding progenitors are termed double negative (DN), due to lack of expression of the co-receptors CD4 and CD8. When T-competent progenitors first enter the thymus, they retain the ability to produce myeloid and natural killer (NK) cells.^66^ Upon receiving Notch and IL-7 stimulation provided by the thymus,^67^ DN cells proliferate and upregulate CD7, CD5, then CD1a, marking the loss of their myeloid and NK differentiation potential.^68–70^

After T-lineage commitment, DN cells proceed to the immature single positive (iSP) stage, characterized by upregulation of CD4.^71^ During this stage, thymocytes become biased toward one of two possible lineages: TCRαβ+ “conventional” T cells, or TCRγδ “innate-like” T cells.^72^ The majority of thymocytes will begin rearranging the TCRβ locus. Thymocytes that produce an in-frame TCRβ rearrangement will express a functional TCRβ protein, which complexes with a surrogate pre-TCRα protein and CD3 to form the pre-TCR complex.^73^ Thymocytes that fail to produce an in-frame TCRβ rearrangement are eliminated through a process termed beta selection. Although pre-TCR expression biases iSPs toward the αβ-T cell lineage, a small subset of iSPs productively rearrange the TCRγ and TCRδ loci, leading to expression of a functional γδ-TCR that initiates commitment to the γδ-T cell lineage.^72^ After pre-TCR expression, αβ-T cell precursors upregulate CD8 to become CD4+CD8+ “double positive” (DP) cells.

Before exiting the thymus cortex, DP thymocytes rearrange their TCRα locus to produce a mature TCRα gene, resulting in expression of the TCRαβ complex on their surface.^74^ Positive selection of thymocytes expressing TCRs that can bind with moderate affinity to self-peptide-human leukocyte antigen (HLA) complexes is critical for further differentiation, as nonreactive TCR clones are eliminated in the cortex through nonselection. During this selection process, DP thymocytes also select a CD8+ cytotoxic phenotype or a CD4+ helper phenotype based on whether the TCR interacts with HLA-I molecules through the CD8 co-receptor, or HLA-II molecules through the CD4 co-receptor.^44^ Next, thymocytes travel to the medulla, where clones expressing TCRs with highly reactivity to self-antigens are eliminated through negative selection.^45^

Mature T cells exit the thymus and enter the circulation as naive T cells, where they scan throughout the body for their cognate antigen. When a naive CD4 or CD8 T cell encounters its target antigen, it undergoes rapid clonal expansion and differentiates into an effector phenotype. CD8 T cells differentiate into cytotoxic T lymphocytes (CTLs), which can directly kill cells presenting the target antigen. CD4 T cells differentiate into a range of “helper” phenotypes, which secrete subsets of cytokines to support the function of CTLs, as well as other innate immune cells. Once the initial infection is cleared, the majority of differentiated effector cells die; however, a subset of effector cells are maintained as memory T cells. Memory T cells are of particular clinical interest, as they are relatively long-lived, but can mount an immune response to their target antigen much more rapidly than a naive T cell.^75^

### Learning from human development to make T cells *in vitro* with stromal feeder cells

Motivated by therapeutic and research applications, scientists have long sought to recreate T cell development *in vitro* (Fig. 1B). Early attempts to differentiate T cells have focused on recapitulating key features of the thymus.^76^ Among the multitude of molecules produced in the thymus, Notch signals are crucial for T cell development and, in combination with the cytokines FLT3L and IL-7, are sufficient to support T cell development from primary hematopoietic stem and progenitor cells (HSPCs) *in vitro.*^77^ Immortalized stromal cell lines engineered to express Notch ligands, such as OP9-DL1 and OP9-DL4 systems, have been used successfully for decades.^77^ Additional feeder-based systems have emerged in recent years, including the artificial thymic organoid (ATO) platform, which more accurately phenocopies some aspects of three-dimensional thymic structure.^78,79^

These feeder-based protocols were originally developed and implemented with primary HSPCs, derived either from cord blood (CB) or bone marrow, as their starting material.^77,79–81^ But these human-donor-derived cell sources suffer similar limitations as primary T cells in terms of scale and intradonor variability. PSCs can theoretically provide a vastly more scalable, cheaper, and genetically consistent source of T cells. A long-standing goal has thus been to produce HSPCs with T lineage potential from PSCs as starting material.

As blood progenitors are derived from the mesoderm, the majority of established PSC-to-T cell differentiation protocols involve a mesoderm induction step. The first *in vitro* blood differentiation protocols tended to phenocopy the early yolk sac-derived primitive wave, and generated mixed populations that lacked robust T lineage potential.^82–84^ This is predominantly due to the addition of exogenous Activin A without Wnt supplementation during the mesoderm induction step, which promotes the development of primitive-biased mesoderm.^85,86^ By manipulating Wnt and activin/nodal signaling, multiple groups succeeded in generating so-called definitive HSPC-like cells with T lineage potential.^87–89^ These PSC-derived HSPCs could develop into mature T cells *in vitro* on stromal feeder layers.

Note that these cells still lack some key attributes of true HSCs as they have thus far failed to engraft and reconstitute immunodeficient mice without extensive genetic manipulation.^90^ This could reflect the fact that these cells more closely resemble an HSC-independent hematopoietic progenitor, or that they are similar to true HSCs but require further maturation in a fetal liver-like environment before they attain engraftment potential.

Despite the remaining hurdle of engraftment, protocol advances in both HSPC differentiation and T cell differentiation have had a profound impact across scientific disciplines. Not only have they served as powerful platforms for studying cellular development and modeling disease, but they also have demonstrated the feasibility of PSC-derived T cell immunotherapies. In a landmark study, Themeli et al. demonstrated that induced PSCs (iPSCs) reprogrammed from a T cell clone could be differentiated into cytotoxic CAR T cells *in vitro.*^91^ Although these PSC-derived T cells had functional properties more akin to γσ, as opposed to αβ-T cells, they were capable of eradicating CD19+ tumor cells *in vitro* and in a xenograft leukemia model.^91^

### Chemically defined T cell differentiation systems

Despite this exciting progress, the aforementioned PSC-to-T cell differentiation protocols all require animal derivatives, including serum, immortalized stroma, or basement-membranes extracted from tumor cultures. Undefined animal derivatives, including sera and extracts, introduce lot-to-lot variability,^92,93^ pose a risk of contamination,^94^ and mask the underlying signals that drive developmental processes. Furthermore, engineered stromal cell lines such as OP9-DL1 present a constant level of Notch ligand over time.^77^ These systems are not ideal for supporting differentiation processes where dynamic changes in signaling strength are required. Collectively these are significant barriers to clinical translation of xenogeneic differentiation processes.

To enable safer, tunable, and robust differentiation protocols, our group and others have focused on building chemically defined methods that avoid feeder cells, serum, and membrane extracts. A major milestone was hit in 2017 when Shukla et al. showed that primary human HSPCs derived from umbilical CB could be directed to become T cell progenitors using recombinant Notch ligands rather than OP9 stroma.^95^ Shukla et al. discovered that the cell adhesion molecule VCAM1 can be used to increase the strength of Notch signaling.^95^ Although immobilizing recombinant DLL4 alone to a culture surface was insufficient to promote T cell progenitor development, the addition of immobilized recombinant VCAM1 synergized with DLL4 to unlock T lineage differentiation potential.^95^ We subsequently extended this method to take CB-derived HSPCs all the way to mature T cell phenotypes using a multistage model-guided media optimization process.^96^

The next key challenge was to extend these chemically defined differentiation protocols to make use of PSCs, rather than CB as starting material. In 2020, Motazedian et al. demonstrated differentiation of CD4+, CD8+ DP, and CD3- cells from iPSCs in feeder-free conditions using an air–liquid-interface culture system.^97^ These cells could subsequently mature into CD3+, TCR+ cells on OP9-DL4 cells.^97^ The cells produced in their culture system express the T-lineage associated recombinase gene surprisingly early in their development.^97^ The authors suggested that these cells represent an HSC-independent T cell progenitor that emerges directly from hemogenic endothelium.^97^

Iriguchi et al. were the first group to differentiate PSCs all the way into mature T cells without the use of immortalized stroma.^98^ The authors reprogrammed iPSC lines from T cells and applied a modified version of previously established chemically defined blood differentiation process to make HSPCs.^87,89^ They transferred these HSPCs on to culture vessels coated with recombinant DLL4 and retronectin, a fragment of the fibronectin protein that can bind to a4b1, the same integrin that interacts with VCAM1.^98^ This process yielded CD8 single positive (CD8SP) cells that expressed the original TCR from the parent iPSC clone. Impressively, these cells were capable of expansion and produced effector cytokines in response to stimulation, demonstrating important functional properties of primary T cells.^98^ They were also capable of specifically killing target cells expressing the cognate peptide antigen of the TCR both *in vitro* and *in vivo.*^98^

Although most of the results presented in the Iriguchi et al. study were generated using a serum-based media, the authors also reported a successful, although less efficient, serum-free process.^98^ This study is an important milestone for clinical translation of PSC-derived T cells, but the method does not appear to be effective for iPSC lines where the TCR locus is in the un-rearranged germline configuration.^98^ This limitation indicates that this protocol fails to capture some key aspect of T cell development.

Less than a year after the Iriguchi et al. study, Trotman-Grant et al. were the first group to report defined differentiation of αβTCR+ DP T cell progenitors starting from PSCs that did not already comprise a rearranged TCR.^99^ These authors generated CD34+ HE cells (CD34 marks both HE cells and HSPCs) and then transitioned them to a downstream T cell differentiation culture system where Notch signals were provided by microbeads functionalized with DLL4.^99^ This process produced TCR+, CD3+, and DP cells at very high purities, with >80% of cells co-expressing CD4 and CD8β and ∼75% of cells co-expressing αβTCR and CD3 by day 42 of differentiation.^99^ A key advantage of this process is the ability to control the timing and dose of Notch signaling by the provision of engineered beads, in a system that may not be limited by two-dimensional surface area.

Our group sought to build on these impressive results and address some important remaining limitations for chemically defined PSC-to-T cell differentiation cell differentiation.^100^ We aimed to overcome the low efficiency of converting PSC-derived CD34+ HSPCs into lymphoid cells and to establish defined differentiation media that could take cells all the way to a αβTCR+, CD8 SP T cell state, the phenotype of functional effector T cells that leave the thymus at the end of development.

The CD34+ cells used as input into downstream T cell differentiation in the Trotman-Grant et al. study have an immunophenotype more consistent with HE, rather than HSPC.^99^ During ontogeny, HE cells first undergo EHT to become HSPC before they enter the thymus. HE cells likely cannot appropriately respond to differentiation conditions meant to support T cell development. To address this missing step, we established a dedicated chemically defined EHT culture system and showed that this improved the efficiency of T-lineage specification by more than an order of magnitude.^100^

We used a multistage model-guided optimization to build an open media formula that efficiently supports CD3+, αβTCR+, CD4+, and CD8+ DP differentiation from PSC-derived HSPCs. We also used αCD3, αCD28, and αCD2 antibody complexes to mimic the TCR:HLA engagement event that drives positive selection in the thymus, allowing us to mature our DP cells into to CD8SPs. These CD8SPs had a conventional immunophenotype and possessed the ability to expand and secrete cytokines in response to stimulation.

Jing et al. have also demonstrated defined differentiation of PSC-derived functional T cells using a similar process.^101^ Jing et al. and Michaels et al. provide independent validation that HSPCs that emerge in the presence of recombinant DLL4 and VCAM1 can mature into functional T cells.^100,101^ The Jing et al. study also showed that knocking down the histone methyltransferase EZH1 can significantly improve the efficiency of T cell differentiation.^101^ As EZH1 is a positive regulator of Notch signaling, this study further emphasizes the importance of carefully titrated Notch activation during T cell development.^101^

After decades of enabling foundational work, the past 2 years have seen a burst of publications in the area of defined T cell differentiation from PSCs, moving this paradigm ever closer to clinical translation.

## Mastering T Cell Development by Controlling the Magnitude and Timing of Key Signaling Programs

Recent advances in chemically defined T cell differentiation systems will facilitate clinical translation of PSC-derived immunotherapies. In addition to reducing the risk of contamination and improving consistency, defined systems are inherently more tunable. This makes them easier to iteratively optimize.

The past few years have also seen an influx in advanced multiomic analyses of human T cell development as it occurs *in vivo.*^102–106^ *In vivo* studies and *in vitro* differentiation experiments have collectively shown that successful T cell differentiation, and the functional properties of the resulting T cells, depend on the timing and magnitude of a small set of key regulatory programs, including Notch-, TCR-, and cytokine-signaling. Defined differentiation platforms afford the unique and timely opportunity to precisely manipulate these key pathways, setting the stage for an era of rapid progress in T cell manufacturing.^96,100,101^

We envision that two foundational approaches will work in concert to realize this vision—bioengineering and synthetic biology. Hereunder, we provide a perspective on some of the most compelling opportunities to advance PSC-derived T cell production by controlling the timing and strength of key signaling programs by engineering the cell-extrinsic niche and cell-intrinsic genetic programs.

### Engineering cytokine signaling to improve differentiation efficiency and reduce cost

T cell development from HSPCs requires activation of a collection of cytokine receptors, initiating a cascade of signal-transduction events that ultimately activate and repress a collection of transcription factor programs that drive cells forward through development. IL-7 and FLT3L are both essential for T cell development.^67,77^ Additional cytokines can enhance or accelerate this process.^96^ The signal transduction cascades and gene expression programs that mediate the effect of these cytokines are both context dependent and share overlapping nodes with other cellular signaling processes.^107^ This makes it extremely difficult to assess the impact of altering the dosage and timing of exogenous cytokines. Getting the dynamic levels of cytokines just right has major implications for the clinical success of T cell differentiation pipelines. The wrong cytokine dosages can drive cells toward alternate fates or completely block differentiation.^96^

### Niche engineering of cytokine signaling

**FIG. 2.**
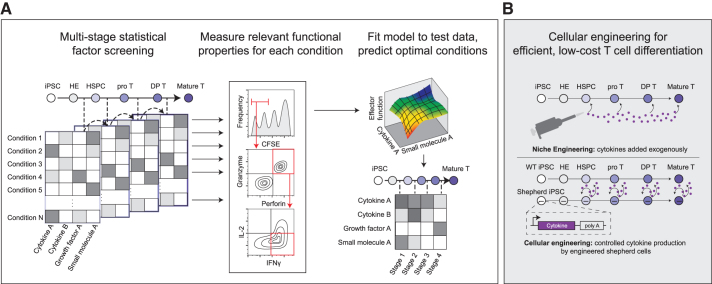
Engineering cytokine signals to enhance *in vitro* T cell differentiation. **(A)** Schematic overview of a general model-guided approach to optimizing the dosage and timing of cytokine exposure required to support functional T cell differentiation. **(B)** Schematic overview of a synthetic biology approach where cytokines are manufactured by the differentiating cells themselves. DP, double positive T cell progenitor; pro T, T cell progenitor.

To address this challenge, we and others have successfully employed statistical design of experiments to rapidly and comprehensively explore the space of cytokine combinations^96,108^ (Fig. 2A). Importantly, we encourage the use of models that can account for changing cytokine requirements over time. By understanding the ancestor–progeny relationships during differentiation, it is possible to apply multistage statistical learning to maximize the yields of intermediate and terminal cell types over the course of a long developmental process.^96^

A typical approach is to use one of several statistical models to choose a limited set of cytokine dosage combinations for a fixed time period in the differentiation process. Cytokines of interest could be manually selected from the literature or identified using high-throughput screens of receptor knockouts or cell–cell signaling interactions.^109^ Yields of a target population are quantified, often using flow cytometry for a set of cell-state defining surface markers. Next, a model is constructed based on the sampled cytokine combinations and extrapolated to the untested parameter space.^96^ Optimal cytokine concentrations are predicted and then experimentally validated for their ability to increase the yield of the target immunophenotype at each interval.^96^

In the future, we propose screening for conditions that maximize the yield of cells with desired functional properties such as target cell killing, cytotoxic cytokine secretion, proliferation, and persistence, as opposed to cell surface phenotypes (Fig. 2A). Ultimately, the goal of T cell immunotherapy is to safely and effectively eradicate cancer. Producing PSC-derived cells with a surface marker expression profile that matches primary T cells is an important first step and making a “T-cell like” product will ease the path to regulatory approval. But it is not clear whether PSC-derived cells that phenotypical resemble conventional αβT cells are actually the best population for immunotherapy.

Alternatively, PSC-derived cells that resemble other lymphoid types such as NK, invariant NK, or γδT cells may be superior in some therapeutic contexts.^110–112^ It is conceivable that the most useful cells might not even show clear resemblance to any primary equivalent. As our clinical knowledge grows, we will find better phenotypical and functional correlates with therapeutic success. These new markers can serve as the goal posts for optimizing *in vitro* differentiation conditions.

### Cell engineering of cytokine signaling

To complement this dynamic media development strategy, synthetic biology approaches could be used to write differentiation programs into the stem cell genome.^113^ Although synthetic biology strategies have not been applied to T cell differentiation extensively as of yet, strategies of interest include “forward programming”—the controlled expression of transcription factors mimicking developmental trajectories;^114^ or engineering the differentiating cells to express and secrete factors that can engage with the cells' endogenous receptor signaling programs to promote differentiation (Fig. 2B).

Because cytokines need to be dosed and timed appropriately for successful differentiation,^96^ crude cytokine overexpression techniques have thus achieved limited success. Recent advances in synthetic biology are enabling precise control of gene expression dosage and timing using small molecule inducers, optogenetics, synthetic receptors, and microRNAs.^115–118^ We recently demonstrated the feasibility of this strategy using a PSC model of gastrulation.

We engineered stem cells to express and secrete BMP4, a factor that is typically added exogenously to promote germ-layer differentiation.^119^ By fine-tuning BMP4 expression levels using synthetic microRNA target sites,^118^ we were able to precisely control the differentiation outcome and completely obviated the need for exogenous BMP4.^119^ As the field makes strides to address the ongoing challenges of large payload delivery,^120^ off-target editing, and transcriptional silencing,^121^ synthetic control over cellular differentiation becomes an increasingly plausible manufacturing strategy.

### Making the right T cells by controlling the magnitude and timing of TCR signaling

Recent progress has enabled efficient differentiation of functional CD8+, effector T cells from PSCs with TCR loci in the germline configuration. However, when the starting PSCs comprise a fully recombined TCR or constitutively express a CAR, these differentiation protocols tend to produce cells with innate-like functional properties instead of conventional αβT cells.^40,91^ A related limitation is that chemically defined differentiation protocols can make CD8+ effector T cells^98,100^ but defined differentiation of functional CD4+ helper T cells has not yet been demonstrated. Both of these issues stem from a common underlying problem—failure to provide the correct timing and magnitude of TCR signaling *in vitro*.

### Overcoming premature TCR-like signals to make conventional T cells

A recent study made the interesting observation that premature TCR signaling (that can be imparted by a pre-rearranged TCR or CAR) can dampen Notch pathway activity.^40^ This increases the threshold of Notch input signals required to promote conventional T cell development. This model also helps to explain the known phenomena that γδT cells, which produce a fully formed TCR earlier in development,^122^ require higher levels of Notch input signals to successfully differentiate.^123^ Thus, carefully managing the complex interplay between Notch and TCR signaling is likely to be very important for efficient *in vitro* differentiation of T cells harboring an engineered TCR or CAR.

Recognizing this phenomenon, the authors elegantly showed that targeting the CAR into the TRAC locus, which delays CAR expression until after beta selection, provides the right timing of complete TCR expression, and makes cells more similar to αβ T cells when coupled with an appropriately strong Notch ligand.^40^ This can further be enhanced by modifying the co-stimulatory domains to get the magnitude of CAR signaling closer to a physiologically relevant range.^40^ Although this is an important step, the resulting T cells still displayed an unconventional phenotype, producing significantly lower levels of effector cytokines compared with primary T cells.^40^

Finding a way to precisely juggle TCR and Notch signaling over developmental time could enable production of conventional cells from iPSCs engineered to express CARs and TCRs. On the side of the TCR signal, this can be achieved by inserting *bona fide* TCRs in the TRAC locus, rather than CARs, matching physiological signaling levels. The timing of CAR activation can be regulated using split-CAR designs; for instance, separating the scFV from the intracellular domains^124^ or separating different modules of the intracellular domain.^125^ These different CAR architectures can be induced to dimerize by the user, enabling specific activation of the CAR at a timepoint of interest during differentiation.

**FIG. 3.**
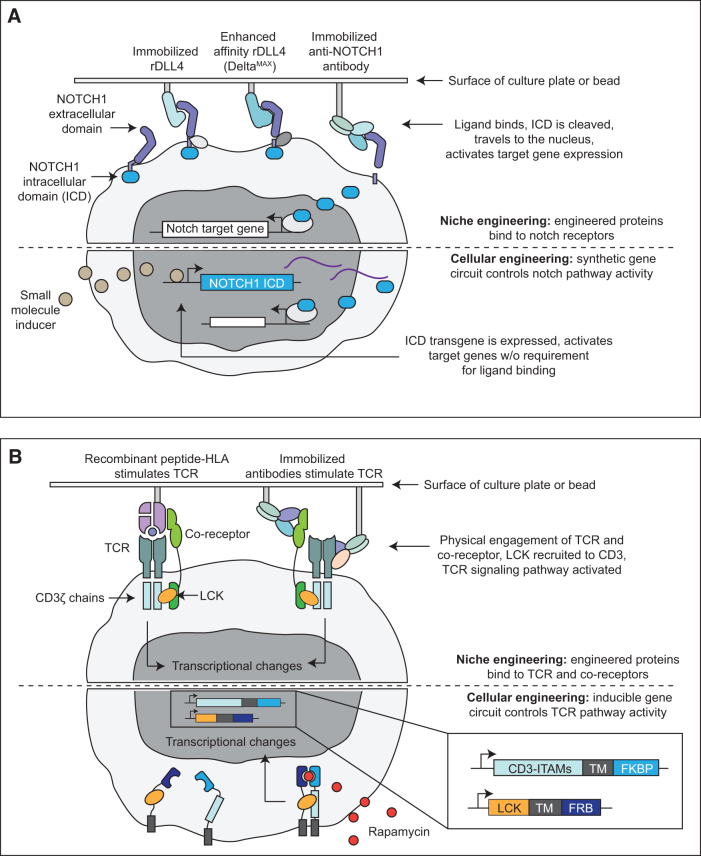
Niche engineering and synthetic biology strategies to precisely control Notch and TCR signaling. **(A)** Methods for manipulating Notch signaling pathway activity. Niche engineering approaches may involve coating plates with immobilized Notch ligands such as DLL4, or anti-Notch receptor antibodies to activate the Notch pathway in developing T cells. Cellular engineering approaches may involve expressing the Notch intracellular domain from a promoter that can be activated by a small-molecule inducer. **(B)** Methods for manipulating TCR pathway activity. Niche engineering approaches may involve coating plates with recombinant peptide-MHC complexes for antigen-specific TCR stimulation, or anti-TCR antibodies for nonspecific TCR stimulation. Cellular engineering approaches may involve fusing CD3 and LCK to domains such as FKBP and FRB, which dimerize in response to the chemical inducer Rapamycin, enabling user-regulated fusion of LCK and CD3 to activate TCR signaling.

On the side of controlling Notch signaling, stronger Notch ligands can be developed through *in vitro* protein evolution, as was recently demonstrated by Gonzalez-Perez et al.,^126^ or through the use of alternate high-affinity binders such as Notch-activating antibodies^127^ tethered to beads or biomaterials (Fig. 3A). Alternately, artificial Notch signals could be provided using synthetic biology approaches that bypass mechanical activation at the cell membrane. For example, overexpressing a constitutively active form of Notch,^67^ or fusing Notch's DNA-binding partner RBPJ to a generic transcriptional activator like VP64 may provide sufficiently strong Notch signaling to counteract the repressive effects of a CAR (Fig. 3A).

### Unlocking CD4 T cell development with niche engineering and synthetic biology

Defined T cell differentiation protocols have thus far failed to generate functional CD4+ helper T cells. CD4+ cells improve the efficacy of CAR T therapy^128,129^ and have applications for treating infection, autoimmunity, and transplant rejection. Thus, producing CD4+ T cells from PSCs has substantial translational potential. Below, we briefly outline the biology underlying CD4 versus CD8 lineage choice, then suggest several strategies by which these biological mechanisms could be leveraged to produce CD4+ T cells *in vitro.*

The decision to adopt a CD4 helper fate versus CD8 effector fate occurs when a TCR expressed by a CD4+, CD8+ DP progenitor engages with a class I or class II HLA molecule. Cells that successfully engage with HLA-I-expressing cells are directed to become CD8SP, whereas TCR engagement with HLA-II promotes differentiation to CD4SP.^130^ CD8 and CD4 are co-stimulatory molecules that enhance downstream TCR signaling. TCR activation downregulates CD8 expression which interrupts signaling for HLA-I restricted cells. In contrast, CD4 expression persists after TCR engagement, leading to an enduring signal in HLA-II restricted T cells.^130^

The duration of TCR signaling during this selection event ultimately dictates whether DP cells become CD8 cytotoxic, or CD4 helper cells.^130^ In current *in vitro* T cell differentiation protocols, positive selection is triggered either by developing cells selecting off of one another,^79^ or using antibodies against the TCR complex.^98,100^ In humans, HLA-II expression is largely restricted to antigen-presenting cells and thymic epithelial cells, whereas HLA-I is expressed broadly across cell types. Thus, cells undergoing *in vitro* differentiation likely have access to HLA-I on other developing T cell progenitors but lack a sufficient source of HLA-II offering one explanation for the lack of CD4+ T cells in current protocols.

One way to provide an appropriate TCR signal to facilitate CD4 differentiation would be to coculture the developing T cell progenitors with primary or immortalized antigen-presenting cells. This is an important advantage of technologies such as the ATO platform,^79^ or differentiation systems that utilize PSC-derived thymic epithelial cells.^131^

Rather than using living material to trigger TCR:HLA-II interactions, recombinant proteins provide a scalable and clinically translatable alternative (Fig. 3B). Soluble or plate-bound recombinant peptide-HLA-II complexes may be sufficient to drive CD4+ fate specification from DP progenitors. But it is not clear how the choice of peptide and HLA-II allele will shape the repertoire of TCRs that can successfully undergo positive selection. A more generalizable approach that would be agnostic to the TCR itself is to use plate-bound antibodies that simultaneously ligate the TCR-constant region and CD4^130^ (Fig. 3B).

Longer-term cell engineering could be used to directly activate downstream TCR signaling. When the TCR engages with MHC, protein tyrosine kinases including Lck are recruited to TCR/CD3 complex.^132^ Lck phosphorylates immunoreceptor tyrosine-based activation motifs (ITAMs) on the cytosolic domain of CD3, which triggers a complex downstream signaling cascade.^132^ It may be possible to construct an inducible protein dimerization system whereby an exogenous small molecule triggers Lck recruitment to the CD3 ITAMs (Fig. 3B). A similar approach was applied to make CAR T activation dependent on a small molecule inducer.^133^

### Avoiding unwanted by-products to increase T cell yield and purity

One compelling opportunity to reduce the cost of *in vitro* T cell manufacturing is to increase the purity and yield of desired target populations at each stage of differentiation. En route to making T cells, protocols first specify PSCs to the mesoderm lineage, then through a HE intermediate, then an HSPC state, next to become T cell progenitors and finally mature T cells.^88^ At every stage, unwanted off-target populations are produced.^88^

**FIG. 4.**
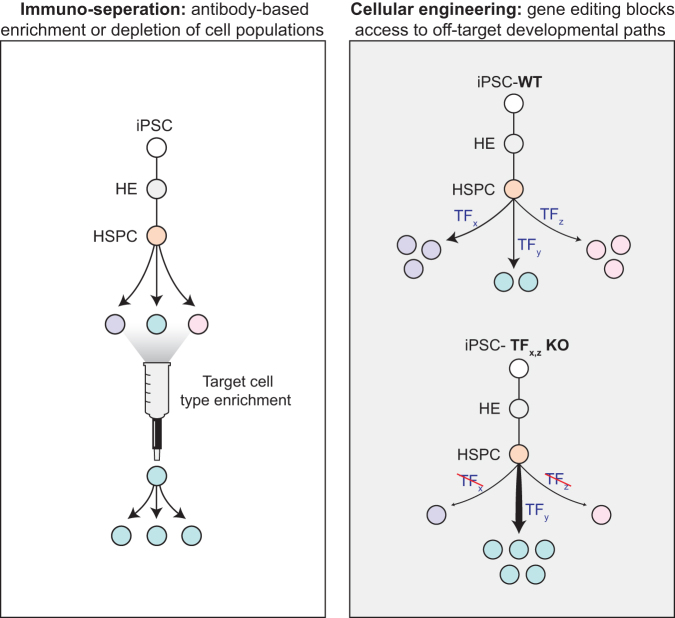
Avoiding unwanted cell populations through affinity-based targeted enrichment and gene editing. Left: Cell populations of interest can be enriched with antibodies through fluorescence-activated cell sorting or magnetic purification. Right: Gene editing can be deployed to block access to undesired cell fates.

These cellular by-products consume resources and limit the maximum yield of the target population per-input PSC. Off-target populations may also compete directly with the desired intermediate.^134^ A proven solution is to enrich for the target population at key transitions in the culture. Exemplifying this approach, we and others routinely select for CD34+ cells early during differentiation by antibody-mediated magnetic separation.^99,100^ Additional enrichment steps, such as at the CD7+ T cell progenitor stage, would likely increase purity and efficiency but would also further complicate the manufacturing workflow and may not reduce the net product cost (Fig. 4).

As an alternative approach, a cellular engineering solution is to block access to unwanted cell fate trajectories by knocking out genes that are required for their development (Fig. 4). Pruning the developmental tree in this manner should allow cell culture resources to be directed efficiently towards supporting T cell development. There is already a strong precedent that knocking out an important fate-specifying gene can improve the efficiency of *in vitro* T cell differentiation. By knocking down EZH1, Jing et al. were able to substantially increase the yield of CD3+ T cell production from PSCs by ∼threefold compared with control conditions.^101^

In addition to blocking access to unwanted developmental paths, cellular engineering can also be applied to enhance or accelerate progression along the target trajectory. Overexpression of hematopoietic transcription factors has enabled or improved *in vitro* differentiation of multiple blood lineages from pluripotent and primary cell sources.^90,135–140^ Although this approach has progressed at a modest pace for the past decade, recent advances in high-throughput perturbation screening technologies are poised to drastically accelerate progress in this space.^141^

## Summary and Conclusion

T cell therapies are disrupting the way we treat disease, but widespread adoption of this modality is limited by our ability to cost-effectively manufacture the right cell types at scale. PSCs are a compelling solution to this challenge that benefit from decades of fundamental research into T cell development using feeder-based differentiation protocols.

Presently, the field is in transitioning to chemically defined bioengineered differentiation systems. Not only are these contemporary approaches more readily translatable to the clinic, but they also afford much more precise control over the levels and timing of signaling inputs during differentiation. T cell development from PSC follows a complex multistage process that is dependent on a carefully balanced orchestra of signals and bioengineering is well suited to accurately reproduce this process *in vitro*.

We anticipate that synthetic biology will drive the next major shift in T cell manufacturing from PSCs. Parallel improvements in our fundamental understanding of T cell differentiation and our ability to synthetically control cell function are setting the stage for more efficient and cost-effective cell therapies. Ultimately, our ability to generate multiple T cell subtypes at scale will enable new therapeutic strategies in oncology and beyond.
